# Buckling Performance of Prefabricated Light-Gauge Steel Frame Materials Under Combined Random Defects During Construction: A CRITIC-Based Analysis

**DOI:** 10.3390/ma18143406

**Published:** 2025-07-21

**Authors:** Gang Yao, Ting Lei, Yang Yang, Mingtao Zhu

**Affiliations:** 1 Key Laboratory of New Technology for Construction of Cities in Mountain Area, Chongqing University, Chongqing 400045, China; yaogang@cqu.edu.cn; 2 School of Civil Engineering, Chongqing University, Chongqing 400045, China; 15859073223@163.com (T.L.); mintz2991@gmail.com (M.Z.); 3 Chongqing Railway Group, Chongqing 400045, China

**Keywords:** light steel frame materials, construction stages, random defects, CRITIC method, weight calculation

## Abstract

Light-gauge steel frame (LGSF) materials are inherently susceptible to stochastic imperfections arising from their design, manufacturing, and erection. These defects can compromise operational integrity and adversely impact structural stability, especially during the construction period. Consequently, a thorough investigation into the buckling characteristics of LGSF materials with such imperfections is imperative. Conventional stochastic probabilistic methods, such as Monte Carlo simulations, often fail to fully capture intrinsic material and complex structural properties, leading to discrepancies between computational predictions and actual behavior. To address these limitations, this study introduces an innovative model using the Criteria Importance Through Intercriteria Correlation (CRITIC) method to assess LGSF materials under combined defects scenarios. The CRITIC method systematically evaluates various buckling modes in LGSFs under combined defects to identify the most detrimental modal combination, representing the most unfavorable scenario. Rigorous finite element analysis is then performed on the LGSF model based on this critical scenario. Compared to conventional approaches, the proposed CRITIC-based combined defects analysis model predicts a 0%~5% reduction in the critical load factor and a 1%~3% increase in ultimate displacement at control nodes. These findings indicate that the CRITIC-based method yields a more critical combination of buckling modes, thereby enhancing the reliability and safety of the simulation results. Furthermore, this research demonstrates that, for LGSF materials, the common assumption that the first-order buckling mode is inherently the most deleterious failure pattern is inaccurate.

## 1. Introduction

Steel has emerged as a principal material in prefabricated construction, owing to its favorable properties, including a high strength-to-weight ratio, standardized production, efficient assembly, and recyclability [1]. Particularly with active promotion through national policies, prefabricated light-gauge steel materials have become a preferred solution for low-rise building construction in rural and township areas [2]. Currently, prefabricated structures suitable for low- to mid-rise residential buildings encompass timber structures, composite wall structures, modular integrated construction, prefabricated reinforced concrete hollow-core shear wall structures, and prefabricated light-gauge steel frames (LGSFs) [3,4,5,6]. Among these, prefabricated LGSF materials are particularly favored in the low-rise building sector, owing to their distinct advantages in modularity, cost-effectiveness, environmental sustainability, and information-integrated manufacturing [7].

However, during fabrication, connection, transportation, and installation, steel materials inevitably develop natural or human-induced defects. These defects can significantly compromise structural safety and serviceability. Particularly in rural and township construction, non-compliant production and installation procedures can readily lead to the degradation of structural performance. Such stochastic defects can induce premature material buckling, thereby posing potential risks to the overall stability and safety of buildings. Consequently, the impact of material defects in light-gauge steel structures on prefabricated LGSFs warrants significant attention [8].

Current research concerning steel containing defects predominantly focus on large-scale complex structures, such as reticulated shells, grid structures, and high-rise steel buildings [9,10,11]. The primary defect types investigated are initial geometric defects and residual stresses. Typically, initial geometric defects manifest in both global and local forms, primarily arising from the initial curvature of structures or members, which exacerbates material perturbation and deformation. Global defects are often simulated via the equivalent load method [12], whereas local defects are commonly modeled using sinusoidal functions [13]. Residual stresses originate from steel production (e.g., hot-rolling or cold-rolling) and fabrication processes (e.g., welding, straightening, reverse bending). Their presence can induce premature elasto-plastic behavior in cross-sections, reduce overall stiffness of materials, and amplify second-order effects. Presently, residual stresses are often analyzed by transforming them into equivalent initial curvatures [14]. Kepple et al. [13] employed the Monte Carlo method to simulate initial geometric thickness, and material defects in cylindrical shell structures, with their results indicating that accounting for such initial defects lowers the axial buckling load of these components. Bielewicz et al. [15] combined the Monte Carlo method with a one-dimensional finite element program to analyze the post-buckling nonlinear behavior of shell structures possessing random geometric defects, assess structural reliability, and validate their method’s efficacy. Sobczyk et al. [16], using the effective stress intensity factor and the weight function method, investigated the influence of random residual stresses on fatigue crack propagation. Cui et al. [17] utilized a synchronous autoregressive model to simulate spatially varying correlated defects in reticulated shell structures and subsequently analyzed their overall structural stability. Kala [18] investigated the deformation of planar trusses with random defects subjected to vertical loading, where findings revealed that asymmetric defects adversely impact the load-bearing capacity of these trusses. Zabojszcza et al. [19] studied the reliability of domes subjected to random defects, employing the first-order reliability method to approximately analyze the impact of nodal position deviations.

In recent years, the focus of the random defect analysis theory has gradually shifted towards general steel members. Vales et al. [20] developed steel beam models using Ansys, incorporating random initial defects and residual stresses, and subsequently analyzed their load-bearing capacity through geometric and material nonlinear analyses. Patton and Singh [21] used finite element analysis to test the compressive performance of concrete steel columns with different interfaces made of LDSS materials. Lauterbach et al. [22] employed random fields to simulate geometric defects, investigating their influence on the buckling loads of steel structures. Domenico [23] investigated slender beam-columns with geometric defects, treating these defects as random fields and analyzing their response within a probabilistic framework. Fina et al. [24] established Gaussian random fields for random defects using probabilistic methods and extended classical probabilistic approaches to encompass fuzzy-random techniques. Mirzaie et al. [25] measured geometric defects generated by probabilistic methods in steel tubes, analyzing defect characteristics stemming from manufacturing processes alongside associated measurement errors. Fonseca [26] considered the influence of initial defects in mechanical property tests of compressed steel columns with different size parameters.

Presently, research methodologies are transitioning from approximate equivalent methods towards stochastic probabilistic approaches, with Monte Carlo simulations being predominant. However, the inherent stochasticity of the stochastic probabilistic method may not fully capture the intrinsic characteristics of materials and structures. This can lead to discrepancies between computational results and actual behavior, thereby compromising the precision of safety assessments for LGSF materials.

With advancements in computational technology, correlation analysis algorithms—effective tools for elucidating relationships between variables and outcomes—have been increasingly applied in civil engineering to facilitate more comprehensive investigations of material properties. Prominent correlation analysis algorithms include Principal Component Analysis (PCA) [27], Multiple Linear Regression (MLR) [28], Stepwise Regression (SR) [29], and the Criteria Importance Through Intercriteria Correlation (CRITIC) method [30]. Hassani et al. [31] demonstrated via simulations that during PCA and multiblock extended consensus PCA, the consumption of degrees of freedom is contingent upon the data’s eigenvalue structure. Wang et al. [30] employed the CRITIC-TOPSIS method to construct an evaluation framework for smart community safety levels, further exploring the weights of contributing factors. Wang et al. [32] proposed the D-MEE model to overcome non-dynamic shortcomings in evaluation models for concrete gravity dams and developed the D-CRITIC method for calculating indicator weights. Diakoulaki et al. [33] introduced a modified CRITIC weighting method that determines weights based on both the variability within each criterion and the conflict between different criteria.

However, the application of correlation analysis methods in civil engineering currently primarily concentrates on concrete materials and structural evaluation models, while research focused on defect analysis in LGSF materials remains relatively scarce [34,35]. For LGSF materials, the combination of different buckling modes plays a decisive role in the stability of LGSF, yet traditional Monte Carlo random distribution simulations are fraught with considerable uncertainty.

To analyze the mechanical characteristics of LGSF materials more accurately, ensuring both the sustainability of their constituent materials and the safety of LGSF buildings, this study introduces the CRITIC-based combined defect analysis model. This innovative approach employs the CRITIC method to assess LGSFs under scenarios of combined stochastic defects. It utilizes the CRITIC method to investigate the buckling mode weights for low-rise prefabricated LGSFs across various construction stages. The aim is to evaluate the extent to which different buckling modes affect system stability. Based on these identified weights of modes, a refined finite element analysis will be conducted on the buckling performance of LGSFs when predominantly influenced by either random global defects or random local defects, respectively. The objective is to determine the most unfavorable operational scenarios for LGSF materials when subjected to random defects at each construction stage, thereby providing a reference for enhancing design and construction safety.

## 2. Finite Element Model of LGSF

### 2.1. Finite Element Model Development and Validation

**Figure 1:**
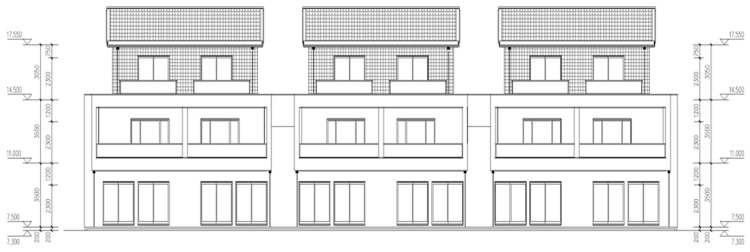
Architectural drawing of LGSFs.

This study focuses on a light-gauge steel frame residential project located in Shangxing, Liyang. The building comprises three layers above ground and one at the basement level, with a typical story height of 3.5 m. Transversely, the building features three spans, totaling 42.35 m. Architectural drawings of the structural model are presented in Figure 1. Service loads, construction loads, and load combination factors for the model were determined by the *Load Code for the Design of Building Structures* [36] and the *Code for Construction of Steel Structures* [12]. Load case combination factors are detailed in Table 1, while the design load values for the model during its construction and service stages are presented in Table 2.

**Figure 2:**
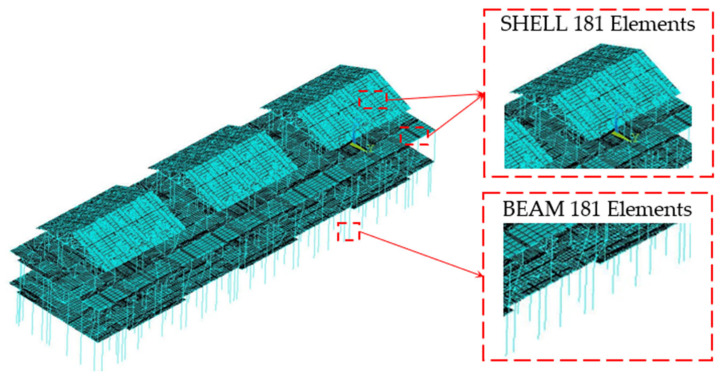
Finite element model of the defect-free LGSF.

This study utilized the general-purpose finite element analysis software ANSYS 2021 for LGSF analysis. Steel (Q235-B) was used for beams, columns, purlins, and bracing. It is an isotropic material following the Von Mises yield criterion, with an elastic modulus of 2.06 × 10^5^ MPa and a Poisson’s ratio of 0.24. Concrete was modeled as an isotropic material with an elastic modulus of 3.0 × 10^4^ MPa and a Poisson’s ratio of 0.2. Beams (B1-B5); columns (Z1-Z3), purlins, and bracing were modeled using BEAM188 elements (a 3D 2-node linear finite strain beam element). Floors and roofs: These were modeled using SHELL181 elements (a 4-node finite strain shell element). The element integration option was set to full integration with incompatible modes to consider both bending and membrane stiffness. Horizontal bracing was modeled using LINK180 elements (a 3D 2-node finite strain link/truss element). SURF154 elements (a 3D structural surface effect element) were overlaid on the SHELL elements to apply various surface loads and effects. The interior partition walls and exterior walls were simplified and applied as equivalent uniformly distributed loads onto the primary beam and column members. This study employs nonlinear finite element equations, which are solved using the arc-length method to conduct a full-range nonlinear analysis of the LGSF. The finite element model of the defect-free system is illustrated in Figure 2.

In finite element analysis, the mesh division strategy for structural members directly influences both accuracy and computational efficiency. To identify an optimal mesh division strategy, thereby enhancing the efficiency and rationality of subsequent analysis, member meshing was categorized during modeling: beam elements were divided into 2, 4, or 6 segments; column elements into 1, 4, or 8 segments; and slab mesh divisions in the X and Y directions were set at ratios of 0.5 and 1 relative to the number of slab-to-beam nodes. To validate the rationality of these mesh divisions, the first-order buckling coefficient of the model and the maximum displacement under static analysis were initially calculated under Permanent Load Design (PLD) conditions. Consequently, by considering 36 mesh division schemes and 4 load cases for the construction stage, a total of 144 defect-free finite element models of the LGSF were established.

**Figure 3:**
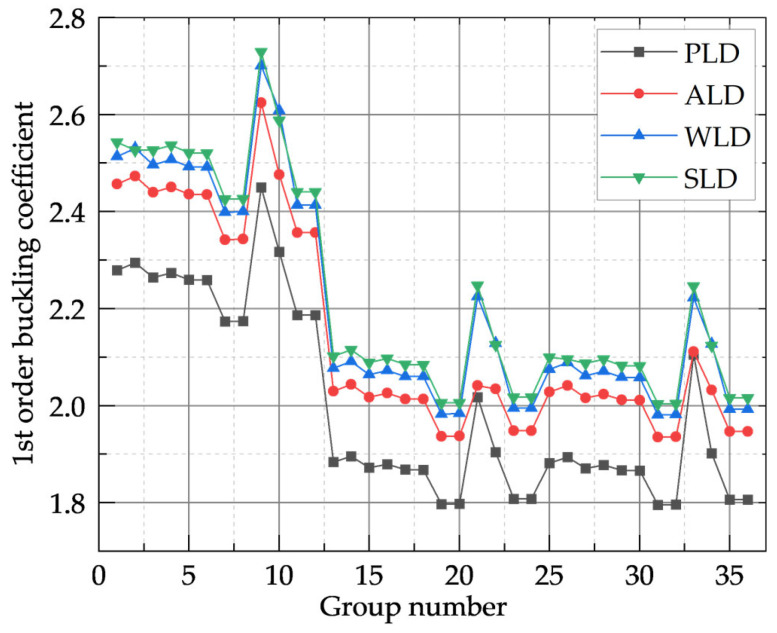
The first-order buckling coefficients for the construction phase under PLD, VLD, WLD, and SLD.

To verify that the selected PLD model configuration was optimal, static and first-order buckling analyses of the defect-free FEM were performed under Variable Load Design (VLD), Wind Load Design (WLD), and Snow Load Design (SLD) conditions using identical mesh divisions and load values, employing a sparse direct solver. The analysis results are presented in Figure 3 and Table 3.

As indicated in Figure 3 and Table 3, under VLD, WLD, and SLD conditions, the variation trend of the first-order buckling coefficient during the construction stage remained broadly consistent across different mesh divisions. Furthermore, the overall variation of the first-order buckling coefficient was also consistent with that observed under PLD. This suggests that for different load combination cases, identical mesh divisions have a minor impact on the evolution of computational results, indicating a degree of stability and accuracy in the calculations. When the column mesh division was set to 1 (groups numbered 1–12), global instability occurred, with all corresponding buckling coefficients exceeding 2. Groups numbered 7 and 8 exhibited relatively small first-order buckling coefficients of 2.17359 and 2.17453, respectively, indicating that the buckling calculation had converged under these conditions. When the column mesh division was not 1 (groups numbered 13–36), the material system experienced local instability. Groups numbered 19 and 20 showed relatively small first-order buckling coefficients of 1.79714 and 1.79765, respectively, suggesting that the buckling calculation had also converged in these cases.

As shown in Table 3, the maximum displacement from the static analysis of the structure during the construction stage under PLD varies between 4 and 7. When the meshing for columns and beams is kept constant, a finer mesh for the slabs in the X and Y directions results in a slight decrease in the maximum displacement, with values fluctuating between 4 and 5. This indicates that slab mesh refinement has a tangible impact on the calculated displacement.

Considering the mesh refinement, the maximum static displacement, and the magnitude of the first-order buckling factor, model 8 (designated as D8) and model 20 (designated as D20) were selected for the analysis of the construction stage.

Table 4 presents the extreme values of the first-order buckling coefficient and static displacement for the defect-free models under PLD, VLD, WLD, and SLD. Under PLD, both the maximum and minimum values of the first-order buckling coefficient were found to be the lowest, indicating that PLD represents the most unfavorable load combination case. Furthermore, the maximum static displacement under PLD was approximately 22% lower than that under WLD, yet about 0.5% higher than those under VLD and SLD, implying that the model produced larger displacements under comparatively smaller applied loads. Considering both the first-order buckling coefficient and static displacement, it was concluded that PLD represents the most unfavorable load combination case for the model, and thus the selection of models D8 and D20 is justified.

**Figure 4:**
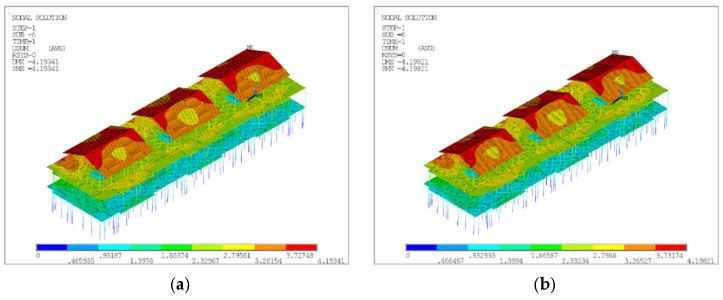
Displacement contours of D8 and D20. (**a**) Displacement contour of D8. (**b**) Displacement contour of D20.

**Figure 5:**
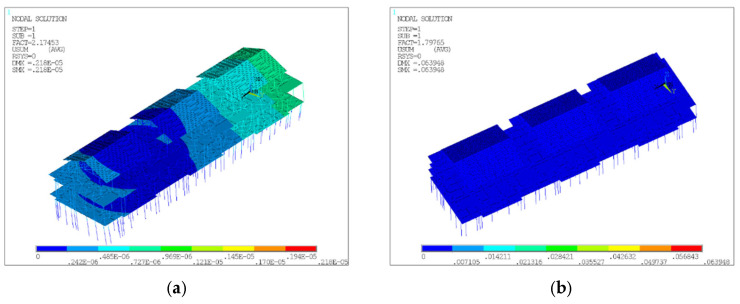
First-order buckling analysis displacement contours for D8 and D20. (**a**) First-order flexural displacement contour of D8. (**b**) First-order flexural displacement contour of D20.

The static analysis displacement contours for models D8 and D20 are presented in Figure 4, and Figure 5 illustrates the first-order buckling analysis displacement contours for models D8 and D20. Figure 5 indicates their respective first-order buckling coefficients to be 2.17453 and 1.79765, with corresponding maximum buckling displacements of 2.18E-06 and 0.063948, respectively. During the construction stage, the model’s first-order buckling coefficient was higher for global instability (D8), while the buckling displacement was larger for local instability (D20). Therefore, model D8 was used to calculate the 1st to 10th order buckling modes to derive the global defect shapes. Model D20 was used to apply the random global defects and the random local defects. 

### 2.2. Combined Defect Application Method

**Figure 6:**
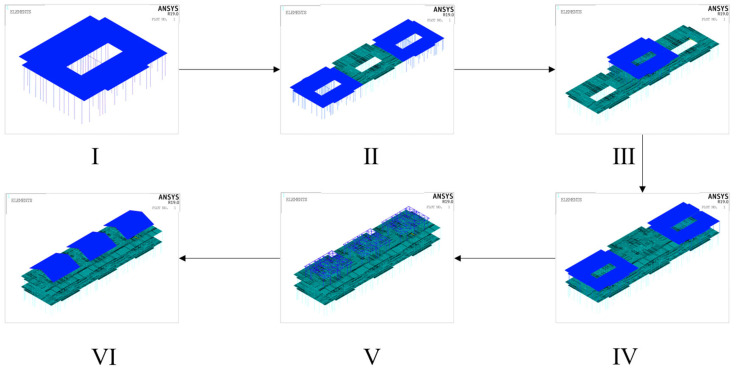
Construction stages of D20.

To investigate the buckling characteristics of the LGSF model under the influence of defects, initial defects (both global and local) were first introduced into the defect-free model D20 using a random defect analysis method based on a modified Monte Carlo approach. To fully account for the stage-dependent buckling characteristics of the model during construction, model D20 was divided into six progressive construction stages, as illustrated in Figure 6. Construction stage I is the erection of the central zone of the first story, stage II is the erection of the peripheral zone of the first story, stage III is the erection of the central zone of the second story, stage IV is the erection of the peripheral zone of the second story, stage V is the erection of beam and column members for the roof level, and stage VI is the completion of the roof structure at the roof level.

**Application of Random Global Defects**. Model D8 was selected for buckling analysis to obtain the 1st- to 10th-order buckling modes of the model under static loading. The nodal displacement results for these 1st- to 10th-order buckling modes of model D8 were batch-extracted to serve as raw data. Subsequently, MATLAB 2021b was used to normalize the extracted raw data. By integrating this normalized data with the modified Monte Carlo-based random defect analysis method, combination coefficients for each buckling mode were determined. This process facilitated the random generation of datasets for the global defects of the LGSF model, as described in Equations (1)–(3). The method of Life and Death Units (LDUs) and MODESH were employed to define the construction stages and adjust the nodal positions in the finite element model. In the actual analysis, to meet analytical requirements while minimizing computational load, 30 sets of random global defect data were extracted for each load case and for each of the construction stages I–VI to perform buckling characteristic analysis. The thus-calculated random global defects were then applied to model D20.

(1) $$\Delta X\prime =\sum_{i=1}^{m}\alpha_{i}\phi_{i}$$

(2) $$r=\max(\left|\frac{\Delta x_{1,\sigma }^{\prime }}{\Delta x_{1}^{\prime }}\right|,\left|\frac{\Delta x_{2,\sigma }^{\prime }}{\Delta x_{2}^{\prime }}\right|,\left|\frac{\Delta x_{3,c\sigma }^{\prime }}{\Delta x_{3}^{\prime }}\right|,\ldots ,\left|\frac{\Delta x_{k,\sigma }^{\prime }}{\Delta x_{k}^{\prime }}\right|)$$

(3) ΔX=r⋅ΔX′

where $\phi_{in}$ represents the displacement vector of the *n*-th node in the *i*-th buckling mode. $\alpha_{i}$ is the combination coefficient, randomly generated via the Monte Carlo method. $\Delta x_{i}^{\prime }$ denotes the value of the structural defect form at node *i*; $\Delta x_{i,cr}^{\prime }$ is the allowable structural defect value at node *i*. *k* is the number of control nodes, and ΔX signifies the amplitude-adjusted global structural defect.

**Application of Random Local Defects**. Model D20 was selected for the application of random local defects to the model. This application is designed to address the impact of initial bending and residual stress on the overall structure. In the random local defect analysis of the model, the sinusoidal function was used for simulation, and the initial maximum displacement amplitude of local members was determined by Equations (4) and (5). Similarly, MATLAB, in conjunction with the Monte Carlo-based random defect analysis method, was used to randomly generate defect displacements along the length of local members. These local defect displacements satisfied the assumption that, in a three-dimensional coordinate model, the defect direction is perpendicular to the member orientation. Analogous to the application of random global defects, 30 sets of defect data were extracted from the randomly generated local defect dataset and applied to the model D20 using LDU and MODESH.

(4) $$\delta =\delta_{\max}\sin(\frac{\pi x}{l})$$

(5) $$P(\delta )=\frac{1}{\sqrt{2\pi \sigma_{1}}}e^{\frac{-{\left(\delta \right)}^{2}}{2\sigma_{1}}}$$

where $\delta_{\max}$ is the defect amplitude, *l* is the member length, *x* is the positional coordinate of a point, and $\sigma_{1}$ is set to $\frac{l}{980}$.

**Application of Random Combined Defects**. To apply random combined defects within the same model, the defect application method required the following adjustments: (1) During the application of random global defects, the positions of the nodes requiring adjustment were modified using a command to select and alter nodal coordinates. (2) Since the nodal positions of the model change after the application of global defects, the displacement changes for other defect locations on a member were calculated only after the member’s defect amplitude and direction had been determined.

In the process of applying random global and local defects, both defect types were scaled down using reduction factors α and β. This resulted in model defect models dominated by either global or local defects, as detailed in Table 5. The defect models dominated by random global defects and random local defects were designated D20_G and D20_L, respectively. A sparse direct solver was then used to calculate the extreme values of the critical load factor and the ultimate displacements at the control nodes for the model under conditions dominated by random global defects and random local defects, respectively.

## 3. CRITIC-Based Combined Defect Analysis Model

### 3.1. CRITIC Method

The CRITIC method is an approach that comprehensively determines objective criteria weights based on two key aspects: the contrast intensity of each criterion and the conflict between criteria. Initially, each criterion is subjected to a normalization process, as detailed in Equations (6) and (7). In calculating the weights for the 1st- to 10th-order buckling modes, the contrast intensity, $S_{j}$, for the *j*-th criterion (buckling mode) quantifies its inherent variability and is expressed by its standard deviation, as shown in Equation (8). A larger standard deviation ($S_{j}$) for a criterion indicates greater variation within its values, thus reflecting higher contrast intensity and resulting in a correspondingly higher weight. The conflict measure for the *j*-th criterion, $R_{j}$, is determined using correlation coefficients with other criteria, as detailed in Equation (9), where $r_{ij}$ represents the correlation coefficient between criterion *i* and criterion *j*. If two criteria exhibit a strong positive correlation, it implies less conflict, which generally contributes to a lower weighting for the criteria involved. The amount of information contained in the *j*-th criterion, $C_{j}$, is calculated as shown in Equation (10). A larger value of $C_{j}$ indicates that the *j*-th criterion carries more relative importance among all the considered criteria. The objective weight of the *j*-th criterion, $\omega_{j}$, is then calculated as the ratio of its $C_{j}$ to the total information content of all criteria, as given by Equation (11). For the CRITIC method, when holding the standard deviation ($S_{j}$), a lower degree of conflict results in a smaller weight. Conversely, a higher degree of conflict leads to a larger weight.

(6) $$\varphi_{ij}^{\prime }=\frac{\phi_{ij}-\min(\phi_{1j},\phi_{2j},\phi_{3j},\ldots ,\phi_{nj})}{\max(\phi_{1j},\phi_{2j},\phi_{3j},\ldots ,\phi_{nj})-\min(\phi_{1j},\phi_{2j},\phi_{3j},\ldots ,\phi_{nj})}$$

(7) $$\varphi_{ij}^{\prime }=\frac{\max(\phi_{1j},\phi_{2j},\phi_{3j},\ldots ,\phi_{nj})-\phi_{ij}}{\min(\phi_{1j},\phi_{2j},\phi_{3j},\ldots ,\phi_{nj})-\max(\phi_{1j},\phi_{2j},\phi_{3j},\ldots ,\phi_{nj})}$$

(8) $$\left\{\begin{array}{c} {\bar{\varphi }}_{j}=\frac{1}{n}\sum_{i=1}^{n}\varphi_{ij}\\ S_{j}=\sqrt{\frac{\sum_{i=1}^{n}{\left(\varphi_{ij}-{\bar{\varphi }}_{j}\right)}^{2}}{n-1}} \end{array}\right.$$

(9) $$R_{j}=\sum_{i=1}^{n}\left(1-r_{ij}\right)$$

(10) $$C_{j}=S_{j}\sum_{i=1}^{n}\left(1-r_{ij}\right)=S_{j}\times R_{j}$$

(11) $$\omega_{j}=\frac{C_{j}}{\sum_{i=1}^{n}C_{j}}$$

### 3.2. Data Preprocessing

The CRITIC method is used to calculate the correlations among the 1st- to 10th-order buckling modes and subsequently derive the respective weights for each mode. To incorporate information regarding the extreme values of the critical load factor, data preprocessing is essential, as detailed in Equations (12)–(15). Initially, the minimum among the critical load factor values within the selected dataset is identified. Subsequently, an amplification factor, $\mu_{\max}$, is determined and applied to scale both the critical load factor values and the buckling mode combination coefficients within each dataset. This process yields modified buckling mode coefficients, $\alpha_{ij}^{\prime }$ (where *i* denotes the *i*-th buckling mode and *j* signifies the *j*-th combination coefficient for that mode), which are thereby enriched with information from the critical load factor values. Through this data preprocessing method, a combined coefficient, $\alpha_{ij}^{\prime }$, is obtained that encapsulates both defect information and information pertaining to the critical load factor values. This combined coefficient $\alpha_{ij}^{\prime }$ then serves as an appropriate data sample for the CRITIC method.

(12) $$f_{\min}=\min(f_{1},f_{2},f_{3},\ldots ,f_{30})$$

(13) $$\mu_{n}=\frac{f_{n}}{f_{\min}}(i=1,2,3,\ldots ,30)$$

(14) $$\mu_{\max}=\max(\mu_{n})(n=1,2,3,\ldots ,30)$$

(15) $$\alpha_{ij}^{\prime }=\mu_{\max}\alpha_{ij}(i=1,2,3,\ldots ,10;j=1,2,3,\ldots ,30)$$

### 3.3. Method Comparison

For the comparative analysis of buckling correlation methods, this study selected established techniques including PCA, MLR, and SR as the comparison methods. A dataset comprising 180 sets of critical load factor values, obtained from the structural system subjected to random global defects, was utilized. These values, in conjunction with their corresponding buckling mode combination coefficients, formed the foundational structure of the trial data samples aimed at validating the applicability and superiority of the CRITIC method.

#### 3.3.1. Preprocessing

In this study, the 180 datasets were categorized into 6 groups according to the construction stage, designated sequentially as S1~S6. The four algorithms—PCA, CRITIC method, MLR, and SR—have distinct data format requirements for their computational processes, necessitating corresponding preprocessing of the data samples. Data preprocessing is exemplified using group S1. When calculating buckling mode weights using the MLR and SR algorithms, the critical load factor, $f_{n}(n=1,2,3,\ldots ,30)$, was treated as the dependent variable, while the 1st- to 10th-order buckling modes, $\phi_{i}(i=1,2,3,\ldots ,10)$, served as independent variables. Here, the $\phi_{i}$ consisted of a 1 × 30 dimensional vector of their respective $\alpha_{ij}$. The data presented in Table 6 served as the trial samples for the MLR and SR algorithms. The data processing approach for the PCA algorithm is identical to that of the CRITIC method, with the processed results shown in Table 7.

#### 3.3.2. Comparison of Trial Results

The data validation results for the PCA method are presented in Table 8. For Bartlett’s test of sphericity, the *p*-value was less than 0.01 within a 99% confidence interval, indicating that Bartlett’s test is valid. However, the KMO value was 0.518, below the prescribed minimum test value of 0.6, suggesting poor partial correlation among the data sample variables. Therefore, the PCA method is not highly suitable for analyzing buckling mode weights in this context.

Table 9 displays the trial results for the MLR algorithm. Although the significance analyses corresponding to the *t*-test and F-test met the requirements (within a 99% confidence interval), Variance Inflation Factor (VIF) values—which indicate the severity of multicollinearity—exceeded 10 (-). This demonstrates strong multicollinearity within the data sample, rendering the MLR algorithm unsuitable for its analysis.

Table 10 presents the trial results for the SR algorithm. The SR algorithm can address data multicollinearity issues and screen for significant variables through statistical tests. Using the SR method, the 6th-, 7th-, 1st-, 10th-, 3rd-, 4th-, and 5th-order buckling modes were identified as the primary variables influencing the critical load factor; VIF values confirmed the absence of multicollinearity among these selected variables. The 6th-order buckling mode had the most significant impact on the system during construction stage I, followed by the 7th- and 1st-order modes, while the 5th-order mode exhibited a negative influence during this stage. The R^2^ value of 0.895, approaching 1, indicated a good fit for this specific stage. However, when the SR method was further applied to fit data for all stages S1–S6 (as shown in Table 11), the mean R^2^ was 0.488, which is less than 0.5, indicating a poor overall fit. Furthermore, as an improvement over MLR, the computational process of SR emphasizes data fitting rather than a direct weighting process. Therefore, the SR method was also deemed unsuitable for this specific weighting task.

Table 12 shows the trial results for the CRITIC method. The CRITIC method can objectively and effectively calculate the weights of each buckling mode, avoiding complex data validation steps while offering computational simplicity. Moreover, the CRITIC weighting process directly assigns objective weights to each buckling mode without interference from other factors, simultaneously considering intra-criterion variability and intercriteria correlations. Consequently, the CRITIC method demonstrates both applicability and superiority for investigating the influence of buckling modes on the buckling characteristics of the LGSF material system.

### 3.4. The Proposed CRITIC-Based Combined Defect Analysis Model

**Figure 7:**
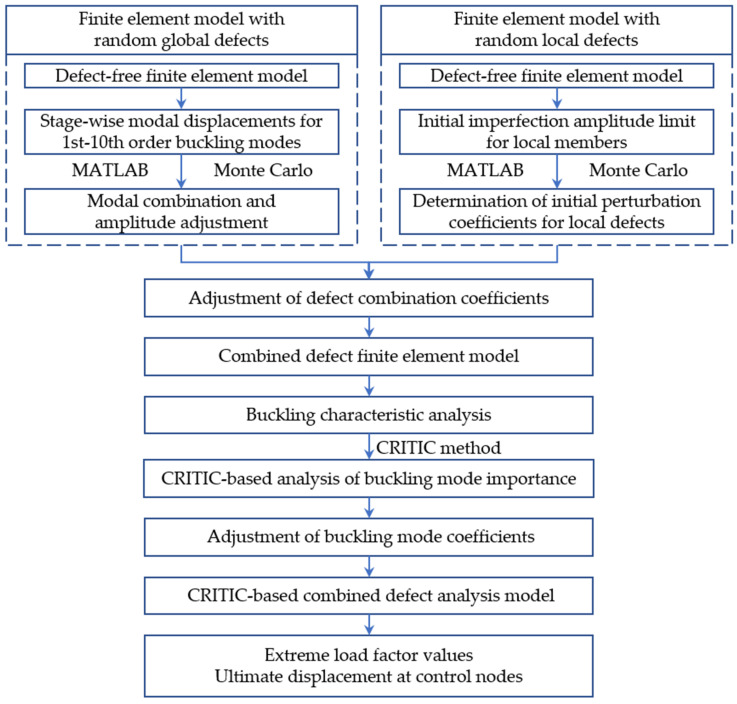
Overall workflow of the proposed method.

The overall workflow of the proposed CRITIC-based combined defect model method is illustrated in Figure 7. Initially, random combined global and local defects are introduced into the established defect-free model using the Monte Carlo method. Subsequently, finite element analysis is performed on this combined defect model to obtain data, including the critical load factor values for the uncorrected model. Subsequently, utilizing the obtained data, the CRITIC method is employed to redefine the weight coefficients for the first 10 buckling modes. This enables a refined assessment of the influence of these buckling mode weights on model stability under combined defect conditions, thereby facilitating the identification of the most unfavorable operational scenarios. Furthermore, these redefined coefficients, $\omega_{ij}$, replace the buckling mode combination coefficients originally generated by the Monte Carlo method, effectively substituting Equation (1) with Equation (16). Finally, once the new modal combination form is established, its amplitude is adjusted. The ANSYS finite element analysis software is then employed for the solution, yielding the final buckling characteristics of the LGSF material model.

(16) $$\Delta X_{i}^{\prime }=\sum_{j=1}^{10}\omega_{ij}\phi_{ij}$$

where *i* denotes the *i*-th construction stage, and *j* denotes the *j*-th buckling mode within the *i*-th construction stage.

## 4. Experiments and Results

Model D20, under the most unfavorable load case PLD, was selected as the subject for the application of random combined defects; it was further divided into six construction stages based on the model’s buckling characteristics. During the application of random global and local defects, reduction factors α was 0.8 and β was 0.5, resulting in LGSF defect models D20_G and D20_L. The CRITIC-based Defect Analysis Model was then employed to calculate the critical load factor values and ultimate displacements at control nodes for the system under conditions dominated by either random global defects or random local defects.

### 4.1. Buckling Mode Importance Analysis

#### 4.1.1. Buckling Mode Importance Analysis Under Dominant Random Global Defects

For each construction stage, this study applied 30 sets of combined defects predominantly characterized by random global defects, generating a total of 180 combined defect models (D20_G). The critical load factors and ultimate displacements at control nodes from these D20_G models were analyzed to assess the model’s buckling characteristics and subsequently determine the buckling mode weights for model D20 across the various construction stages (I–VI).

**Figure 8:**
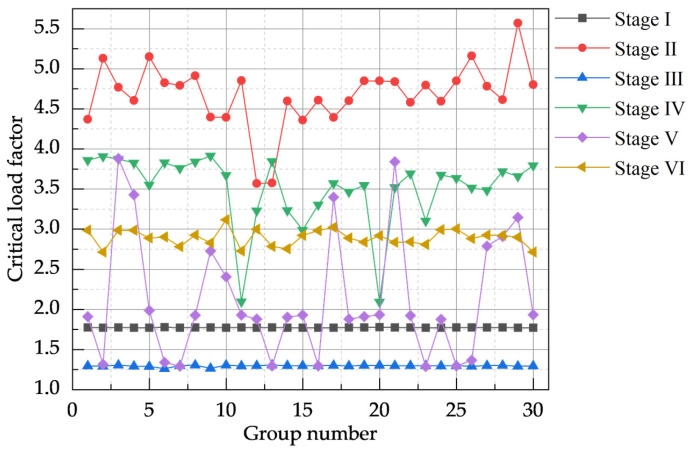
Critical load factor values for a model dominated by random global defects.

The calculated critical load factor results for the D20_G models are presented in Figure 8. During construction stage III, the critical load factor was minimal, with a mean value of 1.295482; conversely, during construction stage II, the model’s critical load factor was maximal, with a mean of 4.672441. The model’s sensitivity to combined defects varied with the construction stage: the critical load factors for stages I and III were relatively insensitive to defect variations, exhibiting small standard deviations (SDs) of 0.0020 and 0.0097, respectively. In contrast, the critical load factors for stages II, IV, and V were significantly affected by combined defects, with corresponding SD values of 0.3940, 0.4470, and 0.7690, respectively.

**Figure 9:**
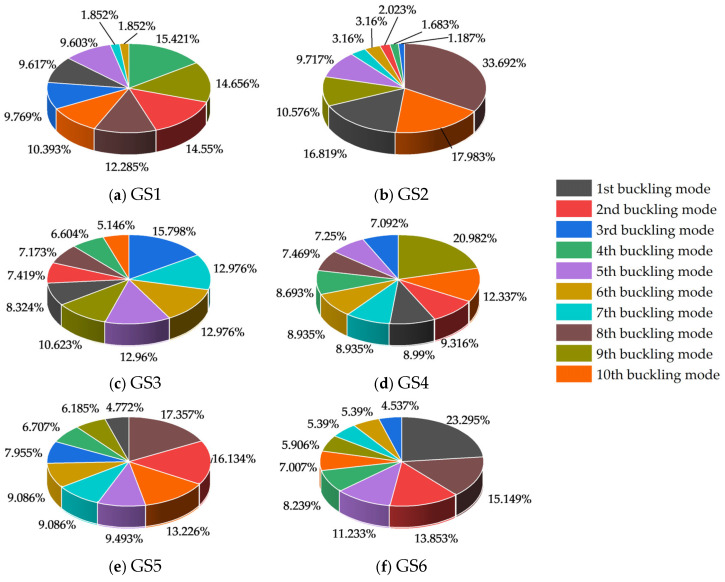
Pie chart of GS1–GS6 buckling mode weights.

Given that the critical load factor under combined defects inherently encapsulates coupled information from global and local defects, the CRITIC method was employed after data preprocessing to assess the importance of each buckling mode. For this analysis, data samples from each construction stage dominated by global defects were sequentially labeled GS1 to GS6, while those dominated by local defects were labeled LS1 to LS6. The CRITIC analysis results for the GS1–GS6 datasets are detailed in Figure 9. This analysis revealed the most unfavorable, controlling buckling modes for the model at each construction stage.

Under GS1, the 4th-order buckling mode had the highest weight (15.42%), while the 6th-and 7th-order modes had the lowest (both 1.85%). Modes with weights exceeding 10% included the 2nd, 4th, 8th, 9th, and 10th. For GS2, the 8th-order buckling mode was dominant (33.69%), and the 3rd-order mode had the minimum weight (1.19%). Modes exceeding 10% weight included the 1st, 8th, 9th, and 10th. In GS3, the 3rd-order buckling mode carried the largest weight (15.80%), with the 10th-order mode being the smallest (5.15%). Modes with weights over 10% were the 3rd, 5th, 6th, 7th, and 9th. For GS4, the 9th-order buckling mode ranked first in weight (20.98%), while the 3rd-order mode was lowest (7.09%). Modes exceeding 10% included the 9th and 10th. In GS5, the 8th-order buckling mode had the maximum weight (17.36%), and the 9th-order mode had the minimum (6.19%). Modes with weights over 10% included the 2nd, 8th, and 10th. For GS6, the 1st-order buckling mode was most significant (23.30%), with the 3rd-order mode having the smallest weight (4.54%). Modes exceeding 10% weight were the 1st, 2nd, 5th, and 8th.

Significant variations were observed in the weights of the most unfavorable buckling modes across stages; the difference between the maximum and minimum weights ranged from 10.65% (GS3) to 32.51% (GS2). This indicates a clear shift in the structure’s susceptibility to specific buckling forms during different construction stages. Therefore, under the influence of combined defects dominated by global defects, the most unfavorable buckling modes for the LGSF material model during construction stages I–VI were identified as the 4th-, 8th-, 3rd-, 9th-, 8th-, and 1st-order modes, respectively.

#### 4.1.2. Buckling Mode Importance Analysis Under Dominant Random Local Defects

To further investigate the impact of combined defects dominated by random local defects on the stability of the D20, this study applied 30 sets of such defects to each of the construction stages I–VI of model D20, generating corresponding combined defect models designated D20_L. Based on the calculated critical load factor values and ultimate displacements at control nodes, the CRITIC method was employed to analyze the importance of the model’s buckling modes. Data samples dominated by local defects were labeled LS1 to LS6 for this analysis.

**Figure 10:**
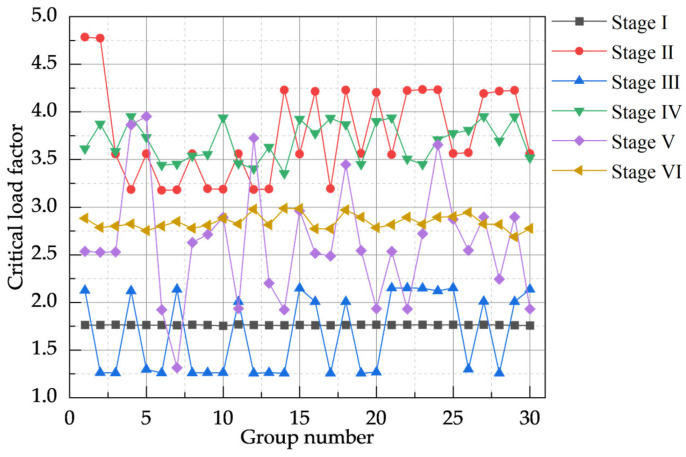
Critical load factor values for models dominated by random local defects.

The calculated critical load factor results for the D20_L are presented in Figure 10. The results show that construction stage III had the lowest mean critical load factor (1.680294), while stage II exhibited the highest (3.76211). Sensitivity analysis of the model to combined defects dominated by local defects indicated that stages I and VI were relatively insensitive to such defect variations, with SD of 0.0028 and 0.0740, respectively; their critical load factors were also comparatively small. In contrast, the critical load factors for stages II, III, and V were more significantly affected by these defect variations, with SD values of 0.4910, 0.4180, and 0.6240, respectively.

**Figure 11:**
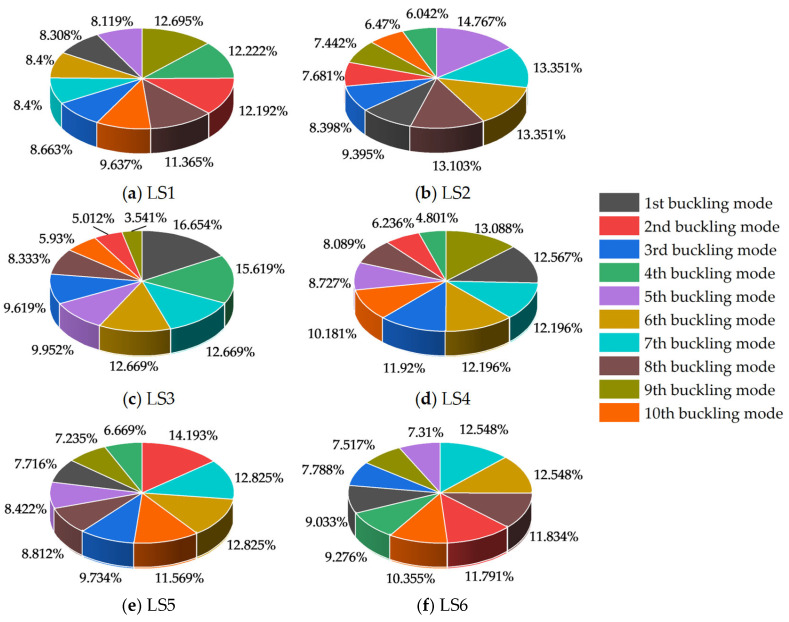
Pie chart of LS1–LS6 buckling mode weights.

Subsequently, the CRITIC method was applied to the LS1–LS6 datasets to identify the key buckling modes at each stage; detailed results are provided in Figure 11. For LS1, the 9th-order buckling mode had the maximum weight (12.70%), while the 5th-order modes had the minimum (8.12%). In LS2, the 5th-order buckling mode ranked first in weight (14.77%), with the 4th-order mode being the lowest (6.04%). For LS3, the 1st-order buckling mode was most prominent (16.65%), while the 9th-order mode had the smallest weight (3.54%). In LS4, the 9th-order buckling mode had the highest weight (13.09%), and the 4th-order mode had the lowest (4.80%). For LS5, the 2nd-order buckling mode carried the maximum weight (14.19%), with the 4th-order mode being the minimum (6.67%). In LS6, the 6th-and 7th-order buckling modes shared the highest weight (both 12.55%), while the 5th-order mode had the smallest (7.31%). Under conditions dominated by local defects, the difference between the maximum and minimum weights of the most unfavorable buckling modes across stages ranged from 4.30% (LS1) to 13.11% (LS3). In summary, for the LGSF subjected to combined defects dominated by random local defects, the most unfavorable buckling modes during construction stages I–VI were identified as the 9th-, 5th-, 1st-, 9th-, 2nd-, and, jointly, the 6th- and 7th-order modes, respectively.

### 4.2. CRITIC-Based Analysis of Buckling Characteristics Under Combined Defects

#### 4.2.1. Critical Load Factor Analysis

**Figure 12:**
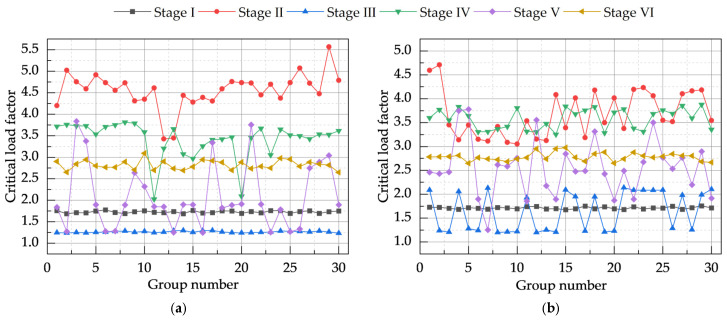
Critical load factor values for CRITIC-based combined defect analysis model. (**a**) Models dominated by global defects. (**b**) Models dominated by local defects.

By incorporating the buckling mode weights obtained in the preceding section into the combined defect model, solution analysis yields the critical load factor values for the model under both global defect-dominated and local defect-dominated scenarios using the CRITIC method, as illustrated in Figure 12. It can be observed that the introduction of the CRITIC method did not significantly alter the variation trend of critical load factors across different defect group numbers for stages I to VI, indicating that the inherent mechanical response characteristics of the system were largely preserved. Table 13 provides a detailed comparison of the critical load factor values for the system under global defect dominance, before and after the application of the CRITIC method. Reallocating weights to different buckling modes via the CRITIC method enables a refined assessment of the material model’s buckling behavior and safety reserves. Specifically, after applying the CRITIC method, the reduction in the minimum critical load factor for each stage ranged from 3.11% (stage III) to 4.77% (stage I). The reduction in the maximum critical load factor for each stage varied between 0.22% (stage III) and 3.84% (stage VI). Notably, the reduction in the minimum critical load factor was generally greater than that of the maximum critical load factor. For instance, in stage I, the minimum critical load factor decreased by 4.77%, whereas the maximum decreased by 0.50%; even in stage VI, where the difference in reduction was relatively small, the decrease in the minimum critical load factor (3.98%) was still slightly higher than that of the maximum (3.84%). This indicates the high sensitivity and effectiveness of the CRITIC method in identifying and quantifying the impact of the most unfavorable defect combinations on the lower bound of the material model’s load-bearing capacity. Therefore, under combined defects dominated by global defects, the defect forms derived using the CRITIC method represent the most unfavorable influence on model stability, yielding more conservative results.

Table 14 details the comparison of extreme critical load factors for the model under local defect dominance, before and after CRITIC method application. After applying the CRITIC method, the minimum critical load factor for each stage, across all construction stages (I to VI), consistently showed a decreasing trend following adjustment by the CRITIC method. The range of this reduction was between 1.65% (stage V) and 4.61% (stage I). Similarly, the maximum critical load factor for all stages also decreased under the CRITIC method, with reductions ranging from 0.06% (stage I) to 4.38% (stage V). For most construction stages (I-IV, and VI), the percentage reduction in the minimum load factor was greater than that in the maximum load factor.

Under dominance by both types of defects, the CRITIC method consistently led to a conservative estimation of the critical load factor, thereby identifying the most unfavorable buckling mode combination weights. Moreover, in most cases, the impact of the CRITIC method on the minimum critical load factor was systematically greater than its effect on the maximum value. This further underscores the potential of the CRITIC method in revealing underlying risks and enabling refined assessments, while also highlighting the sensitivity of its results to the analytical conditions and parameters.

#### 4.2.2. Control Node Ultimate Displacement Analysis

(1) Global defect-dominated model

**Figure 13:**
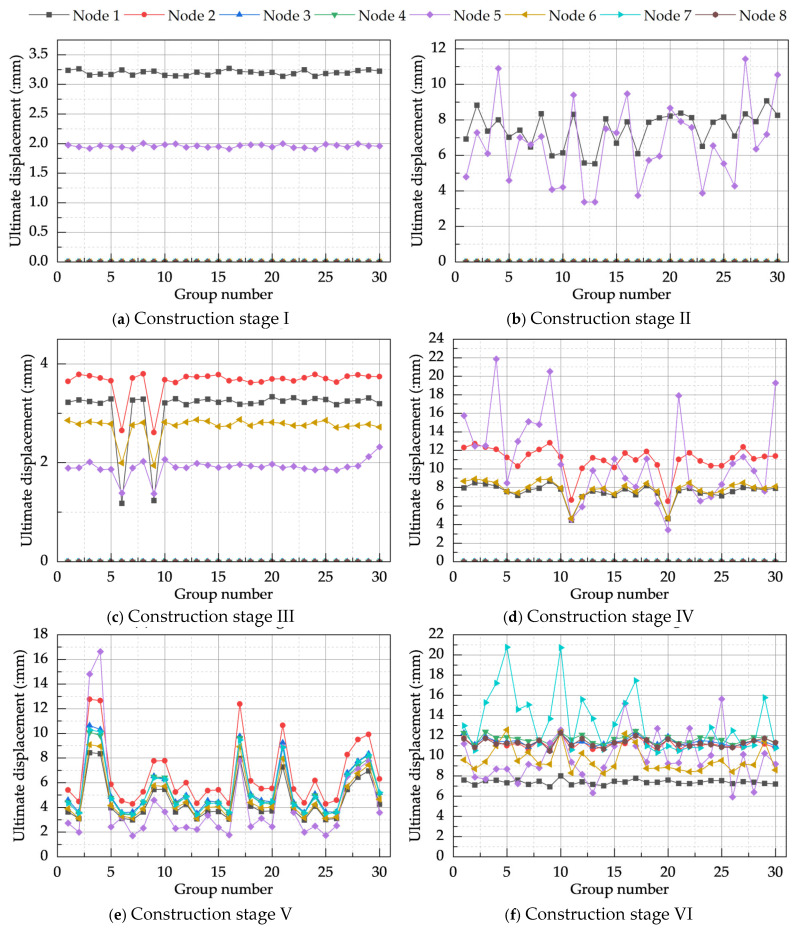
Ultimate displacements of the CRITIC-based global defects-dominated model.

Through finite element analysis, the ultimate displacements at eight control nodes for the model under global defect dominance, based on the CRITIC method, were obtained, as illustrated in Figure 13. Nodes 1–4 correspond to the points of maximum displacement on the first floor, second floor, third-floor beam level, and roof level, respectively; Nodes 5–8 correspond to the points of maximum displacement at the mid-height of the first-story columns, second-story columns, third-story columns, and roof-level columns, respectively.

As shown in Figure 13, nodal displacements during stages I and II were minor and localized, consistent with the characteristics of single-story construction. During stages III–IV, the ultimate nodal displacements began to exhibit significant variations, indicating that the construction had entered a structurally sensitive period, with Node 5 showing considerable fluctuations. In stage III, the displacement of Node 6 exceeded that of Node 5, whereas stage IV demonstrated the opposite outcome. This was attributed to the smaller scale of the second-story columns in stage III, which had not yet formed complete lateral stiffness, thus resulting in larger lateral displacements. Conversely, in stage IV, the complete second-story structure was established, leading to effective control of horizontal displacement. As construction progressed, multiple monitored nodes became active, reflecting the gradual completion and stiffening of the overall structural model. Upon completion of construction in stage VI, the displacements at control nodes became more stable compared to preceding stages, indicating that the model had achieved a stable load-resisting configuration.

Table 15 illustrates the changes in the extreme ultimate displacements at control nodes before and after CRITIC method correction; these displacements increased across all construction stages by amounts ranging from 2.15% to 3.18%. The most significant increase occurred at Node 6 during stage V. Consistent with the analysis in Section 4.2.1, under conditions of smaller critical load factors, an increase in the ultimate displacement at control nodes signifies a reduction in model stability. This implies that under combined defects, the buckling mode combinations determined by the CRITIC method are indeed the most detrimental to model stability. Consequently, the most unfavorable buckling modes for the system during construction stages I–VI were identified as the 4th-, 8th-, 3rd-, 9th-, 8th-, and 1st-order modes, respectively.

(2) Local defect-dominated model

**Figure 14:**
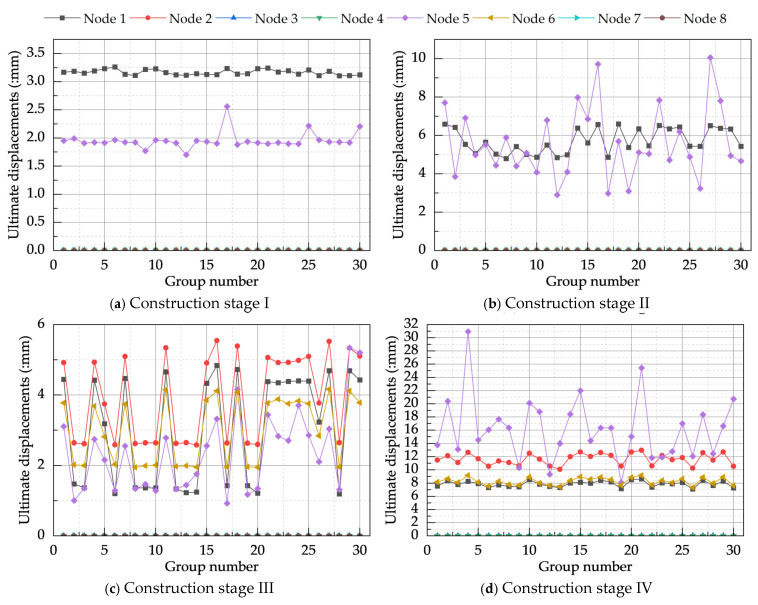

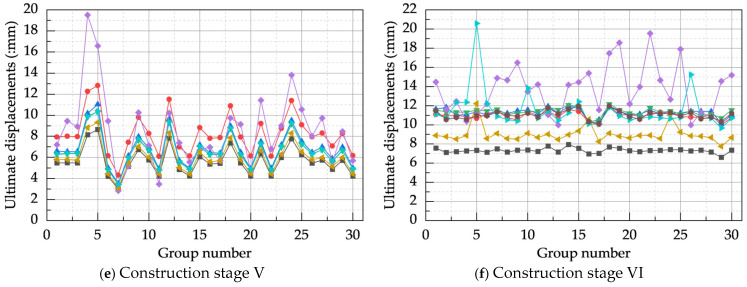
Ultimate displacements of the CRITIC-based local defects-dominated model.

Through finite element analysis, the ultimate displacements at eight control nodes for the model under local defect dominance, based on the CRITIC method, were obtained, as illustrated in Figure 14. Similar to the displacement variations observed under global defect dominance, nodal displacements in stage I were relatively uniform without excessive fluctuations. In stage II, due to the increased scale of the columns, significant variations in displacement occurred across different defect group numbers. Stages IV-VI all exhibited variation characteristics similar to those observed under global defect dominance. Differing from the global defect-dominated scenario, however, system displacements under local defect dominance in stage III showed substantial fluctuations, indicating that the overall structure is more sensitive to local defects during this particular stage.

Table 16 illustrates the changes in the extreme ultimate displacements at control nodes before and after CRITIC method correction; these displacements increased across all construction stages by amounts ranging from 1.98% to 3.17%. The most significant increase occurred at Node 1 during stage VI. Consistent with the analysis in Section 4.2.1, under conditions of smaller extreme load factors, an increase in the ultimate displacement at control nodes signifies a reduction in system stability. This implies that under combined defects, the buckling mode combinations determined by the CRITIC method are indeed the most detrimental to system stability. Consequently, under local defect dominance, the most unfavorable buckling modes for the system during construction stages I–VI were identified as the 9th-, 5th-, 1st-, 9th-, 2nd-, and, jointly, the 6th- and 7th-order modes, respectively.

## 5. Conclusions

This study, contextualized by an actual engineering project, established a finite element analysis model for LGSF materials subjected to combined defects. Through a comparison of four algorithms, an optimization analysis method for buckling mode combinations based on the CRITIC method was proposed. By optimizing buckling mode combinations using the CRITIC method, the most unfavorable buckling mode combination forms for the LGSF at each construction stage were determined. A CRITIC-based combined defect analysis model was developed to analyze the buckling characteristics of the LGSF under the most unfavorable scenarios, dominated by either global or local defects. The following conclusions were drawn:

(1) Compared to PCA, MLR, and SR algorithms, the CRITIC method is more conducive in quantitatively analyzing the importance of the buckling modes in LGSF models. It was found that under combined random defects, the influence of these buckling modes on the model’s buckling characteristics varies across different construction stages. Buckling mode weights typically fluctuated between 5% and 20%. This demonstrates that for LGSF materials, the traditional assumption that the first-order buckling mode is the most unfavorable is inaccurate; analyses simulating model defects based solely on the first-order buckling mode yield unconservative results.
(2) Compared to random defect models established using a modified Monte Carlo-based random defect analysis method, defect models developed using the CRITIC method exhibited lower critical load factors and correspondingly higher ultimate displacements at control nodes. For combined defects under different dominant defect types, the reduction in critical load factors ranged from 0 to 5%, while the increase in ultimate displacement at control nodes ranged from 1 to 3%. This indicates that the combined defect model established using the CRITIC method represents the most unfavorable conditions.
(3) For the engineering case study herein, under combined defects dominated by global defects, the most unfavorable buckling modes during construction stages I–VI were the 4th-, 8th-, 3rd-, 9th-, 8th-, and 1st-order modes, respectively. Under combined defects dominated by local defects, the most unfavorable buckling modes for these stages were the 9th-, 5th-, 1st-, 9th-, 2nd-, and, jointly, the 6th- and 7th-order modes, respectively.

In this study investigating the buckling characteristics of LGSF materials under combined defects, the following areas for potential improvement were identified: (1) Conduct more in-depth research on determining the most unfavorable forms of local defects. The present study identified the most unfavorable local defects through data comparison, based on an initial understanding of the most unfavorable global defects. Future work could build upon this foundation to refine the research methodology, enabling a more accurate consideration of the impact of local defects on the LGSF system during buckling analysis. (2) Perform dynamic response analysis of LGSF under random defects. This paper primarily focused on the buckling stability of such models, with a lack of research into their performance under dynamic loading conditions. (3) The analysis of residual stress defects in this study was based on code provisions, incorporating them into the initial imperfection amplitudes of model members. Subsequent research could refine the finite element model to represent these defects more rationally, leading to more accurate buckling analysis results.

## Figures and Tables

**Table 1:** Load case combination factors.

Load Case	Permanent Load	Variable Load	Wind Load	Snow Load
PLD (Permanent load design)	1.35	0.7	0.6	0.7
VLD (Variable load design)	1.2	1.4	0.6	0.7
WLD (Wind load design)	1.2	0.7	1.4	0.7
SLD (Snow load design)	1.2	0.7	0.6	1.4

**Table 2:** The design load values for the system.

Category	Section Size (mm)	Load Value		
		Standard Value	Service Stage	Construction Stage
I-section column (Z1)	259 × 107 × 6 × 9	78.5 kN/m^3^	-	-
Square hollow section column (Z2)	75 × 75 × 4.5	78.5 kN/m^3^	-	-
Square hollow section truss column (Z3)	60 × 60 × 2.5	78.5 kN/m^3^	-	-
I-section beam(B1)	250 × 125 × 6 × 9	78.5 kN/m^3^	-	-
I-section beam(B2)	250 × 125 × 6 × 9	78.5 kN/m^3^	-	-
I-section beam(B3)	250 × 125 × 3.2 × 4.5	78.5 kN/m^3^	-	-
C-section beam(B4)	150 × 50 × 50 × 4.0	78.5 kN/m^3^	-	-
Double C-section truss beam(B5)	100 × 500 × 20 × 2.5	78.5 kN/m^3^	-	-
Exterior wall	-	9.6 kN/m^3^	-	-
Interior partition wall	-	0.54 kN/m^3^	-	-
Floor grid	-	0.4 kN/m^3^	-	-
Roof	-	0.55 kN/m^3^	-	-
Roof live load	-	-	0.5 kN/m^2^	0.5 kN/m^2^
Floor live load	-	-	2.0 kN/m^2^	0.6 kN/m^2^
Wind load	-	-	0.3 kN/m^2^	0.3 kN/m^2^
Snow load	-	-	0.25 kN/m^2^	0.25 kN/m^2^

**Table 3:** Results of 36 sets of model trial calculations of a defect-free finite element model under PLD in the construction phase.

Group Number	Beam Mesh Division	Column Mesh Division	Floor Y-Direction Mesh Division	Floor X-Direction Mesh Division	Maximum Displacement	First-Order Buckling Coefficient
1	2	1	0.5	0.5	6.27452	2.27920
2	2	1	0.5	1	6.22487	2.29427
3	2	1	1	0.5	4.19172	2.26424
4	2	1	1	1	4.17472	2.27343
5	4	1	0.5	0.5	6.70931	2.25983
6	4	1	0.5	1	6.53670	2.25899
7	4	1	1	0.5	4.22604	2.17359
8	4	1	1	1	4.19341	2.17453
9	6	1	0.5	0.5	6.70584	2.44998
10	6	1	0.5	1	6.55545	2.31677
11	6	1	1	0.5	4.09863	2.18687
12	6	1	1	1	4.14017	2.18703
13	2	4	0.5	0.5	6.28060	1.88360
14	2	4	0.5	1	6.23104	1.89587
15	2	4	1	0.5	4.19643	1.87219
16	2	4	1	1	4.17950	1.87965
17	4	4	0.5	0.5	6.71548	1.86849
18	4	4	0.5	1	6.54293	1.86768
19	4	4	1	0.5	4.23088	1.79814
20	4	4	1	1	4.19821	1.79765
21	6	4	0.5	0.5	6.71209	2.01752
22	6	4	0.5	1	6.56184	1.90396
23	6	4	1	0.5	4.10337	1.80803
24	6	4	1	1	4.14500	1.80791
25	2	8	0.5	0.5	6.28062	1.88183
26	2	8	0.5	1	6.23106	1.89409
27	2	8	1	0.5	4.19645	1.87043
28	2	8	1	1	4.17952	1.87789
29	4	8	0.5	0.5	6.71550	1.86674
30	4	8	0.5	1	6.54295	1.86593
31	4	8	1	0.5	4.23090	1.79545
32	4	8	1	1	4.19822	1.79596
33	6	8	0.5	0.5	6.71211	2.10549
34	6	8	0.5	1	6.56186	1.90214
35	6	8	1	0.5	4.10338	1.80634
36	6	8	1	1	4.14501	1.80621

**Table 4:** Analysis of extreme static displacement and first-order buckling coefficient for defect-free LGSF.

Load Case	First-Order Buckling Coefficient		Extreme Displacement	
	Maximum	Minimum	Maximum	Minimum
PLD	2.44998	1.795451	6.71550	4.09863
VLD	2.62473	1.93496	6.69296	3.93503
WLD	2.70053	1.98086	8.63738	6.13039
SLD	2.72941	2.00359	6.68101	3.87817

**Table 5:** Defect models dominated by random global defects and random local defects.

Model Designation	Defect Type	Reduction Factor
D20_G	Global defect	α
	Local defect	β
D20_L	Global defect	β
	Local defect	α

**Table 6:** Data samples for trial calculation of MLR and SR algorithms.

	$\alpha_{ij}$	1	2	3	4	5	6	7	8	9	10
$f_{n}$											
1.7758		0.0377	0.1288	0.1586	0.0605	0.0224	0.0918	0.0304	0.0241	0.2512	0.1944
1.7739		0.1282	0.2881	0.0689	0.0060	0.0679	0.0195	0.0118	0.1414	0.1338	0.1345
1.7768		0.0854	0.1536	0.0912	0.2074	0.0622	0.1316	0.0925	0.0386	0.0374	0.1001
1.7744		0.0713	0.0921	0.0858	0.0650	0.2363	0.1155	0.0261	0.0606	0.1100	0.1374
1.7716		0.0210	0.0496	0.0656	0.0643	0.1777	0.0062	0.0339	0.1290	0.2247	0.2280
1.7810		0.1184	0.0106	0.1665	0.1527	0.0009	0.2102	0.1055	0.0509	0.0309	0.1532
1.7743		0.1084	0.0032	0.0550	0.1095	0.1536	0.0085	0.1484	0.0739	0.1056	0.2339
1.7779		0.0893	0.1085	0.0279	0.1289	0.1109	0.2034	0.2063	0.0708	0.0257	0.0284
1.7772		0.1903	0.0391	0.0265	0.2129	0.1079	0.0934	0.1120	0.0327	0.0289	0.1563
1.7754		0.1188	0.0109	0.0747	0.2675	0.0690	0.0194	0.0085	0.2000	0.0454	0.1857
1.7754		0.1311	0.1385	0.0156	0.0849	0.0473	0.0936	0.0764	0.1153	0.2670	0.0303
1.7754		0.1894	0.0743	0.1200	0.0949	0.0851	0.0110	0.1272	0.1736	0.0175	0.1070
1.7770		0.2634	0.0747	0.1249	0.0958	0.0424	0.0247	0.0995	0.0252	0.0635	0.1858
1.7744		0.0529	0.1231	0.2358	0.0759	0.0422	0.0050	0.1994	0.1686	0.0524	0.0447
1.7754		0.0026	0.0298	0.1992	0.0325	0.0946	0.1114	0.1316	0.0607	0.2279	0.1097
1.7737		0.0962	0.0037	0.0064	0.1477	0.1885	0.0246	0.1322	0.2500	0.0416	0.1090
1.7739		0.0298	0.0862	0.1138	0.2567	0.1682	0.0312	0.1277	0.0879	0.0179	0.0804
1.7751		0.1678	0.2934	0.1825	0.0420	0.0493	0.0569	0.0164	0.0231	0.0600	0.1086
1.7775		0.1769	0.0591	0.1102	0.0435	0.0718	0.1350	0.1452	0.1638	0.0857	0.0088
1.7805		0.0439	0.0490	0.0682	0.0052	0.0115	0.1858	0.3435	0.1050	0.0472	0.1406
1.7756		0.0396	0.2224	0.2050	0.0663	0.0292	0.1113	0.0564	0.2033	0.0351	0.0315
1.7752		0.0690	0.2183	0.1137	0.0628	0.0924	0.1524	0.0249	0.0356	0.1986	0.0323
1.7741		0.1474	0.2221	0.1710	0.0318	0.2086	0.0616	0.0216	0.0382	0.0362	0.0614
1.7737		0.0440	0.1405	0.1065	0.0833	0.0571	0.0360	0.0014	0.3403	0.0513	0.1396
1.7754		0.1791	0.1568	0.0588	0.0049	0.0306	0.2628	0.0142	0.2693	0.0165	0.0069
1.7747		0.0827	0.0035	0.0262	0.0480	0.0420	0.0266	0.2278	0.2543	0.2510	0.0380
1.7771		0.1246	0.2073	0.0735	0.0346	0.0527	0.2080	0.0587	0.1511	0.0082	0.0813
1.7772		0.0498	0.1648	0.1531	0.0991	0.0391	0.1437	0.2012	0.1408	0.0000	0.0084
1.7746		0.1082	0.0706	0.0416	0.1485	0.1104	0.0285	0.0820	0.0774	0.1416	0.1914
1.7743		0.0021	0.1694	0.0887	0.0749	0.0001	0.0062	0.2161	0.0505	0.1905	0.2016

**Table 7:** Trial data sample applicable to PCA and CRITIC methods.

	i	1	2	3	4	5	6	7	8	9	10
j											
1		0.0399	0.1884	0.2214	0.0090	0.3699	0.2039	0.2039	0.2499	0.0632	0.0798
2		0.4678	1.0413	0.0143	0.2546	0.4798	0.0580	0.0580	0.0543	0.3969	0.4079
3		0.1227	0.1981	0.0488	0.2725	0.2201	0.2389	0.2389	0.1722	0.3568	0.2435
4		0.1783	0.1505	0.0716	0.0024	0.2077	0.3875	0.3875	0.2425	0.4067	0.3212
5		0.1030	0.0412	0.1067	0.0935	0.1869	0.2157	0.2157	0.0825	0.2048	0.0790
6		0.3258	0.1730	0.0397	0.1687	0.0899	0.0089	0.0089	0.2057	0.4442	0.0220
7		0.1087	0.0938	0.0600	0.1028	0.1065	0.3141	0.3141	0.0927	0.3169	0.3178
8		0.3035	0.0189	0.0700	0.2663	0.1252	0.1564	0.1564	0.1727	0.3509	0.1284
9		0.2550	0.1011	0.0238	0.5300	0.2836	0.0344	0.0344	0.1852	0.4675	0.1102
10		0.2887	0.2332	0.1312	0.0541	0.3422	0.2294	0.2294	0.0359	0.1110	0.5187
11		0.4380	0.0643	0.1414	0.0797	0.2411	0.0125	0.0125	0.1161	0.5391	0.3149
12		0.4494	0.0466	0.1099	0.0585	0.2509	0.0343	0.0343	0.1925	0.2988	0.0781
13		0.1117	0.2574	0.5959	0.0408	0.4747	0.0041	0.0041	0.1385	0.6033	0.6416
14		0.0482	0.0518	0.3495	0.0832	0.2176	0.2754	0.2754	0.1787	0.0084	0.3095
15		0.3589	0.2191	0.2824	0.3979	0.0512	0.0882	0.0882	0.5776	0.8738	0.5678
16		0.0376	0.1555	0.2052	0.8188	0.0406	0.1067	0.1067	0.1748	0.8800	0.6281
17		0.4136	0.6872	0.3295	0.0628	0.3620	0.1429	0.1429	0.2984	0.4642	0.3365
18		0.2637	0.0160	0.0755	0.0352	0.1792	0.2179	0.2179	0.0588	0.2351	0.3708
19		0.0258	0.0008	0.0029	0.0666	0.1873	0.2025	0.2025	0.0270	0.2000	0.0984
20		0.0462	0.4304	0.3472	0.0050	0.3701	0.2618	0.2618	0.1851	0.4724	0.4894
21		0.1328	0.4665	0.1329	0.0042	0.2360	0.4043	0.4043	0.2584	0.0722	0.5411
22		0.5137	0.7009	0.4284	0.1320	0.1255	0.2252	0.2252	0.3688	0.7660	0.6768
23		0.1116	0.4409	0.1984	0.0920	0.5855	0.1447	0.1447	1.0250	0.8408	0.4288
24		0.4109	0.2785	0.0176	0.1544	0.3823	0.6669	0.6669	0.3647	0.5410	0.5744
25		0.2007	0.1503	0.1284	0.0530	0.4420	0.0666	0.0666	0.4078	0.0857	0.6185
26		0.1891	0.2880	0.0144	0.0542	0.2285	0.3687	0.3687	0.0403	0.3953	0.2680
27		0.0509	0.2039	0.1550	0.0616	0.2454	0.2436	0.2436	0.0208	0.3978	0.3888
28		0.2925	0.0704	0.0806	0.2824	0.2260	0.0748	0.0748	0.1733	0.2789	0.1227
29		0.0698	0.4427	0.0853	0.0392	0.6567	0.0001	0.0001	0.2902	0.1262	0.0976
30		0.0399	0.1884	0.2214	0.0090	0.3699	0.2039	0.2039	0.2499	0.0632	0.0798

**Table 8:** Trial calculation results of PCA.

KMO Test and Bartlett’s Test of Sphericity		
KMO value	0.518	
Bartlett’s test of sphericity	$X^{2}$	947.578
	d*f*	45
	*p*	0.000 ***

Note: *** denote significance at the 1% levels.

**Table 9:** Trial calculation results of MLR.

Independent Variable	Regression Coefficient	*p*	t	F	VIF	R^2^
Constant	1.614	0.000 ***	11,290.908	19.438 (*p* = 0.000 ***)	-	0.897
1st-order buckling	0.167	0.000 ***	76.696		-	
2nd-order buckling	0.155	0.000 ***	79.882		-	
3rd-order buckling	0.164	0.000 ***	69.766		-	
4th-order buckling	0.163	0.000 ***	74.58		-	
5th-order buckling	0.149	0.000 ***	67.702		-	
6th-order buckling	0.176	0.000 ***	90.733		-	
7th-order buckling	0.167	0.000 ***	97.711		-	
8th-order buckling	0.155	0.000 ***	100.848		-	
9th-order buckling	0.156	0.000 ***	90.985		-	
10th-order buckling	0.163	0.000 ***	71.408		-	

Note: *** denote significance at the 1% levels.

**Table 10:** Trial calculation results of SR.

Independent Variable	Regression Coefficient	*p*	t	F	VIF	R^2^
6th-order buckling	0.02	0.001 **	9.254	26.887 *p* = 0.000 **	1.433	0.895
7th-order buckling	0.011	0.001 **	6.165		1.23	
1st-order buckling	0.011	0.001 **	4.712		1.158	
10th-order buckling	0.008	0.002 **	3.422		1.407	
3rd-order buckling	0.008	0.005 **	3.129		1.255	
4th-order buckling	0.007	0.003 **	3.274		1.129	
5th-order buckling	−0.007	0.014 **	−2.673		1.292	

Note: ** denote significance at the 5% levels.

**Table 11:** Analysis of S1–S6 buckling mode influence using the SR algorithm.

Number	Retained Variable	Regression Coefficient	*p*	t	F	VIF	R^2^
S1	6th-order buckling	0.02	0.000 **	9.254	26.887 (*p* = 0.000 **)	1.433	0.895
	7th-order buckling	0.011	0.000 **	6.165		1.23	
	1st-order buckling	0.011	0.000 **	4.712		1.158	
	10th-order buckling	0.008	0.002 **	3.422		1.407	
	3rd-order buckling	0.008	0.005 **	3.129		1.255	
	4th-order buckling	0.007	0.003 **	3.274		1.129	
	5th-order buckling	−0.007	0.014 **	−2.673		1.292	
S2	4th-order buckling	−3.518	0.001 ***	−3.939	8.353 (*p* = 0.006 **)	1.045	0.432
	1st-order buckling	−2.952	0.006 ***	−3.005		1.035	
	3rd-order buckling	−2.282	0.038 **	−2.185		1.08	
S3	3rd-order buckling	0.041	0.038 **	1.804	2.813 (*p* = 0.012 **)	2.082	0.262
	6th-order buckling	0.022	0.024 **	1.203		2.003	
	7th-order buckling	0.018	0.03 **	1.063		1.931	
S4	9th-order buckling	−2.752	0.008 **	−2.875	6.142 (*p* = 0.026 **)	1.003	0.713
	7th-order buckling	−2.115	0.040 **	−2.162		1.003	
S5	1st-order buckling	5.501	0.036 **	2.198	4.829 (*p* = 0.036 **)	1	0.117
S6	10th-order buckling	0.684	0.008 ***	2.851	8.894 (*p* = 0.009 **)	1.073	0.506
	7th-order buckling	−0.514	0.014 **	−2.632		1.014	
	4th-order buckling	0.548	0.029 **	2.314		1.059	

Note: *** and ** denote significance at the 1% and 5% levels, respectively.

**Table 12:** Trial calculation results of CRITIC method.

Buckling Mode	Contrast Intensity	Conflict Measure	Information Content	Weight (%)
1st-order buckling	0.153	8.565	1.311	9.08
2nd-order buckling	0.241	7.204	1.737	12.031
3rd-order buckling	0.143	8.111	1.156	8.007
4th-order buckling	0.18	9.384	1.691	11.707
5th-order buckling	0.152	8.975	1.368	9.47
6th-order buckling	0.15	8.627	1.298	8.985
7th-order buckling	0.15	8.627	1.298	8.985
8th-order buckling	0.201	7.433	1.494	10.345
9th-order buckling	0.243	7.162	1.737	12.029
10th-order buckling	0.204	6.623	1.352	9.36

**Table 13:** Comparison of critical load factor values for the global defect-dominated model.

Stage	Minimum			Maximum		
	Traditional	CRITIC		Traditional	CRITIC	
I	1.77006	1.685551	(−4.77%)	1.77787	1.769057	(−0.50%)
II	3.56872	3.40220	(−4.67%)	5.57023	5.366507	(−3.66%)
III	1.26132	1.222039	(−3.11%)	1.30647	1.303577	(−0.22%)
IV	2.09414	1.998511	(−4.57%)	3.91295	3.866979	(−1.17%)
V	1.28968	1.240389	(−3.82%)	3.87876	3.771617	(−2.76%)
VI	2.71184	2.603972	(−3.98%)	3.11541	2.995883	(−3.84%)

**Table 14:** Comparison of critical load factor values for the local defect-dominated model.

Stage	Minimum			Maximum		
	Traditional	CRITIC		Traditional	CRITIC	
I	1.7546	1.6737	(−4.61%)	1.7665	1.7654	(−0.06%)
II	3.1764	3.0308	(−4.58%)	4.7847	4.6524	(−2.77%)
III	1.2559	1.2012	(−4.36%)	2.1522	2.1193	(−1.53%)
IV	3.3555	3.2552	(−2.99%)	3.9517	3.8765	(−1.90%)
V	1.3135	1.2918	(−1.65%)	3.9529	3.7797	(−4.38%)
VI	2.6889	2.5943	(−3.52%)	2.9859	2.9569	(−0.97%)

**Table 15:** Comparison of ultimate displacements under dominant random global defects.

Node	I		II		III		IV		V		VI	
	Traditional	CRITIC	Traditional	CRITIC	Traditional	CRITIC	Traditional	CRITIC	Traditional	CRITIC	Traditional	CRITIC
1	3.125	3.198	7.315	7.493	3.037	3.114	7.286	7.506	4.451	4.547	7.216	7.395
2	-		-	-	3.541	3.638	10.737	11.024	6.518	6.687	10.903	11.172
3	-		-	-	-	-	-	-	5.363	5.508	11.104	11.389
4	-		-	-	-	-	-	-	5.212	5.345	11.383	11.642
5	1.914	1.956	6.427	6.612	1.856	1.906	10.673	10.937	4.248	4.345	9.501	9.761
6	-		-	-	2.667	2.735	7.605	7.797	4.687	4.836	9.354	9.589
7	-		-	-	-	-	-	-	5.249	5.378	12.865	13.236
8	-		-	-	-	-	-	-	-	-	11.015	11.276

**Table 16:** Comparison of ultimate displacements under dominant random local defects.

Node	I		II		III		IV		V		VI	
	Traditional	CRITIC	Traditional	CRITIC	Traditional	CRITIC	Traditional	CRITIC	Traditional	CRITIC	Traditional	CRITIC
1	3.101	3.167	5.614	5.725	2.981	3.041	7.687	7.879	5.527	5.655	7.091	7.316
2	-	-	-	-	3.860	3.958	11.355	11.645	8.138	8.249	10.679	10.972
3	-	-	-	-	-	-	-	-	6.642	6.804	10.991	11.282
4	-	-	-	-	-	-	-	-	6.417	6.566	11.110	11.363
5	1.913	1.955	5.444	5.583	2.326	2.388	15.846	16.176	8.376	8.500	13.392	13.705
6	-	-	-	-	2.920	2.996	8.098	8.275	5.794	5.968	8.804	9.009
7	-	-	-	-	-	-	-	-	6.447	6.607	11.227	11.507
8	-	-	-	-	-	-	-	-	-	-	10.753	11.038

## Data Availability

The raw data supporting the conclusions of this article will be made available by the authors on request.
